# Computational Homogenisation and Identification of Auxetic Structures with Interval Parameters

**DOI:** 10.3390/ma18194554

**Published:** 2025-09-30

**Authors:** Witold Beluch, Marcin Hatłas, Jacek Ptaszny, Anna Kloc-Ptaszna

**Affiliations:** 1 Faculty of Mechanical Engineering, Department of Computational Mechanics and Engineering, Silesian University of Technology, Konarskiego 18A, 44-100 Gliwice, Poland; 2 Independent Researcher, Księdza Pawła Janika 15B/27, 41-806 Zabrze, Poland; 3 Faculty of Mechanical Engineering, Department of Engineering Materials and Biomaterials, Silesian University of Technology, Konarskiego 18A, 44-100 Gliwice, Poland

**Keywords:** auxetic material, numerical homogenisation, identification, interval parameters, directed interval arithmetic, response surfaces, finite element method

## Abstract

The subject of this paper is the computational homogenisation and identification of heterogeneous materials in the form of auxetic structures made of materials with nonlinear characteristics. It is assumed that some of the material and topological parameters of the auxetic structures are uncertain and are modelled as interval numbers. Directed interval arithmetic is used to minimise the width of the resulting intervals. The finite element method is employed to solve the boundary value problem, and artificial neural network response surfaces are utilised to reduce the computational effort. In order to solve the identification task, the Pareto approach is adopted, and a multi-objective evolutionary algorithm is used as the global optimisation method. The results obtained from computational homogenisation under uncertainty demonstrate the efficacy of the proposed methodology in capturing material behaviour, thereby underscoring the significance of incorporating uncertainty into material properties. The identification results demonstrate the successful identification of material parameters at the microscopic scale from macroscopic data involving the interval description of the process of deformation of auxetic structures in a nonlinear regime.

## 1. Introduction

The design of new structural materials is a significant contemporary engineering challenge. Inhomogeneous materials represent a substantial and continually evolving group of materials, as they may possess properties that are not available to homogeneous structural materials. Consequently, their application in a variety of industries, including mechanical, automotive, marine, and aerospace, has become extensive. Materials in this category encompass a wide range of structures, including particle and fibre composites, layered structures, foams, and lattice structures, among others [1].

An interesting group of inhomogeneous materials are auxetic structures, which are characterised by a negative Poisson’s ratio. Due to their unique geometry and mechanical properties, auxetic structures find applications in a wide range of fields, from automotive and medical equipment to sports equipment and environmental protection [2,3]. Given the complexity of their geometry, 3D printing is the most suitable manufacturing method, as it allows for the precise reproduction of details while minimising material waste [4]. Auxetic structures are usually classified according to their geometry and deformation mechanism [4,5]. The most common types of structure are re-entrant (RE) honeycomb, chiral structures, arrowhead structures, and rotating polygon structures [5,6]. Re-entrant honeycomb structures are made of concave, inverted cell elements with inclined ribs pointing ‘inward’ (inverted honeycomb geometry). These structures are highly anisotropic. Compared to classical honeycombs, they have a higher Young’s modulus and a higher shear modulus; that is, they are stiffer and more resistant to lateral deformation. Chiral structures consist of elements (e.g., bars and ribs) connected at different angles and forming spiral or circular patterns. Due to their unusual geometry, the mechanical properties of such structures can be directionally dependent. Arrowhead structures consist of repetitive elements arranged in the form of triangular arrow-shaped cells that form a grid with a characteristic arrangement. In addition to auxetic properties, these structures often have good compressive and tensile strength. Other common auxetic structures include antichiral structures in which elements are connected in such a way that they form oppositely twisted (antichiral) patterns. These patterns are formed by connecting ‘nodes’ or points of contact using bars or ribs, arranged around a central point, but with opposite twist directions on either side [6]. Auxetic structures, characterised by high anisotropy and a favourable stiffness–weight ratio, are widely used in automotive applications [7,8]. Research in this area is focused primarily on energy absorption during crashes, which increases the safety of vehicle operation. An interesting example is a new crash box composed of an aluminium shell and a 3D-printed lattice core [7]. The structure designed in this way contributes to improving the energy absorption efficiency of the new crash box and increases durability and safety during vehicle operation. The article [8] proposes a new car side door beam using an auxetic structure that significantly improves side impact resistance compared to conventional beams. Auxetic materials effectively dampen noise and vibration, which can contribute to improved driving comfort [9]. They can also be used in smart sensors and adaptive components that respond to driving conditions [10]. In the medical field, auxetic structures can act as strong and flexible scaffolds to support tissue growth and regeneration [11,12,13,14,15]. They improve the cushioning properties of implants, resulting in a reduction in microinjuries and increased patient comfort [15]. The paper [16] presents a study of a mesh consisting of antichiral cells made using 3D printing technology. When PLA material has shape memory, the size and shape of the mesh can be adjusted to fit different surfaces. The mechanical properties of the mesh can be adjusted by changing the angle of the arc. It is shown that changes in arc angle significantly affect the range of Poisson’s ratio adjustment. For configurations θ_1_ = 180° and θ_2_ = 60°, the Poisson ratio can drop to as low as -5, indicating a strong auxetic effect. The grid is adjusted to different surfaces, including different parts of the human body. A device is also developed on this basis for monitoring electromyographic (EMG) signals. The paper [17] discusses biofabrication’s role in tissue engineering, focusing on 3D-printed auxetic scaffolds that mimic native tissue properties. The authors emphasise the significance of selecting appropriate structural parameters, particularly in the filament intersection zones. Due to their excellent energy damping properties and low weight, auxetic materials are used in sports equipment, including helmets, protective clothing, and sports shoes [18,19]. In one study [20], auxetic foam was used for the last layer (comfort) in a sports helmet. The study showed that helmets with auxetic foam inserts dampened linear accelerations significantly better than their conventional counterparts, especially in lateral impacts and high energies. The effectiveness of auxetic foam may be due to its ability to densify on impact and resist ‘bottoming out’, making it a promising material for the design of modern sports helmets to protect against brain injuries. Athletic footwear uses soles with auxetic layers. An example is Nike Free RN Flyknit Shoes, with an engineered closed-cell foam sole with a structure of rotating auxetic triangles [21]. The soles of the shoes stretch laterally and longitudinally during the wearer’s movement, improving grip and cushioning. Auxetic structures are also used to protect trees. In one paper [22], a wire auxetic protective structure was developed and studied, which adapts to the tree trunk and promotes the regeneration of damaged bark. With a negative Poisson’s ratio, the structure expands synchronously with the trunk, distributing the pressure evenly and preventing further damage. Simulations have shown that this solution is much safer and more effective than traditional wire mesh, which can damage the tree. In addition, auxetic reinforced composites are suitable for use as armour panels in vehicles, bulletproof vests, and various other applications that require impact protection [23,24]. A brief review of the contemporary applications of auxetic structures suggests that these materials have significant potential for increasing use. To enhance the efficiency of developing new structures, it is essential to adopt appropriate modelling approaches that account for the uncertainty in their parameters.

Macroscopic mechanical behaviour in inhomogeneous materials is directly influenced by their microscopic characteristics [25]. The experimental identification of microscopic parameters involves a range of microscopy techniques, depending on the type of material and the research objective. Scanning electron microscopy (SEM) and transmission electron microscopy (TEM) enable the analysis of auxetic structures and have been widely used to evaluate surface morphology, beam geometry, and microcracks in foams, composites, and 3D-printed structures, as demonstrated in the works [26,27,28,29,30]. X-ray microtomography (micro-CT) allows three-dimensional visualisation of cells and internal deformations in auxetic foams and hybrid auxetics [31,32]. Confocal laser scanning microscopy (CLSM) has been used for the precise observation of fibre cross-sections, deformation analysis, and verification of the geometry of 3D-printed scaffolds [17,33]. The combined use of these microscopic techniques allows for a comprehensive assessment of the microstructure, deformation behaviour, and defects in auxetic materials, which is essential for the design and optimisation of structures with tailored mechanical properties.

One key aspect of multiscale modelling is the ability to analyse inhomogeneous materials at the microscale. The determination of the macroscopic, mechanical, or other properties of inhomogeneous materials can be achieved using homogenisation methods. One of the most versatile homogenisation methods is computational homogenisation, which is highly efficient due to its ability to analyse complex structures with linear and nonlinear constitutive relationships [34,35]. Computational homogenization applies numerical approaches to solve boundary-value problems (BVPs), capturing the mechanical behaviour of materials at a designated scale. Such methods usually include the finite element method (FEM) [36,37] and the boundary element method (BEM) [38,39,40]. Moreover, the formulation and solution of the inverse problem allow the topology and microstructure parameters to be identified based on the macroscopic properties of the homogenised material [41,42]. In order to solve this identification problem, it is necessary to employ optimisation methods. Since the objective function (or functions) in inverse tasks is often multimodal and/or discontinuous, global optimisation methods (e.g., evolutionary algorithms) are commonly applied to these problems [43,44]. The homogenisation procedure assumes certain deterministic values of the input properties, but manufacturing processes and measurement errors can cause uncertainty [45,46], which makes the calculation of macroscopic parameters unreliable. Therefore, developing computational approaches that account for uncertainties is critical for the efficient design and production of advanced materials [47,48]. In the context of uncertainty, the following three distinct categories of uncertainty have been identified: stochastic uncertainty, incertitude uncertainty, and ignorance [49]. Incertitude uncertainty is common in engineering problems where the distribution of a variable is not known. Incertitude uncertainty can be represented using intervals and fuzzy sets [50,51]. From an information processing perspective, this representation of quantities is also referred to as ‘information granularity’ [52,53]. Computational homogenisation is usually a time- and resource-consuming process. This is especially disadvantageous when global optimisation methods, which process a set of potential solutions, are employed [43,54]. In order to reduce the computational effort, a range of metamodels may be employed. Metamodels encompass a range of approaches, including polynomial response surfaces [55], radial-basis functions [56], Kriging [57], and artificial neural networks (ANNs) [58].

In this paper, computational homogenisation and computational inverse homogenisation of selected auxetic structures are performed. Some material and topological parameters are assumed to be imprecise (incertitude uncertainty) and are, thus, described using interval numbers. Furthermore, the material nonlinearity of the auxetic structure is assumed. To reduce computational effort, response surfaces in the form of ANNs are employed as metamodels.

## 2. Applied Methods

### 2.1. Directed Interval Arithmetic

Interval numbers make it possible to represent uncertainty in cases where only a range of admissible parameter values is available. In interval arithmetic, a single number is expressed as an interval $\bar{a}$ [59], as follows:

(1) $$\bar{a}=\left[a^{-},a^{+}\right]=\left\{a\in \bar{a}:\;\;a^{-}\leq a\leq a^{+}\right\}$$

where *a*^−^ and *a*^+^ ∈ ℝ are the left and right ends of the interval $\bar{a}$.

When both ends are equal, the interval becomes degenerate. Classical interval arithmetic is founded on the rules of arithmetic defined for real numbers, which are subsequently generalised to interval quantities. The set of basic operations on intervals includes addition, subtraction, multiplication, division, multiplication by a scalar, and inversion of an interval [60]. However, the classical framework is limited by the absence of operations that act as inverses to addition and multiplication [61]. Consequently, when interval arithmetic is applied to systems of equations, the resulting interval bounds tend to widen, which leads to growth in uncertainty [62]. This effect can be mitigated to a significant extent by employing modifications of classic interval arithmetic, such as generalised interval arithmetic [63], segment analysis [64], and directed interval arithmetic [65]. In this paper, directed interval arithmetic is used as the most versatile and efficient technique among those mentioned.

A directed interval number is an ordered pair of real numbers, as follows:

(2) $$\bar{a}=\left[a^{-},a^{+}\right]=\left\{a\in \bar{D},{\;a}^{-},a^{+}\in R,D=P\cup I\;\right\}$$

where P and I are, respectively, the set of all proper ($a^{-}<a^{+}$) and all improper ($a^{-}\geq a^{+}$) interval numbers with real ends [65].

Directed interval arithmetic encompasses all the basic arithmetic operations: addition, subtraction, multiplication, and division. In addition, it defines new operators [66], as follows:

- Opposite of addition:
  (3) $$\forall \bar{a},\in D{-}_{D}\bar{a}=\left[-a^{-},-a^{+}\right]$$
- Inverse of multiplication:
  (4) $$\forall \bar{a}\in D\backslash Z\;{1/}_{D}\bar{a}=\left[{1/a}^{-},-1/a^{+}\right]$$
  where the set $Z=Z_{P}\cup Z_{I}$ contains all directed intervals with element 0, as follows:
  (5) $$\begin{array}{c} Z_{P}=\left\{\bar{a}\in P:a^{-}\leq 0\leq a^{+}\right\}\\ Z_{I}=\left\{\bar{a}\in I:a^{+}\leq 0\leq a^{-}\right\} \end{array}$$
  Based on the above, directed interval arithmetic implements additional operations, as follows:
- Directed subtraction:
  (6) $$\forall \bar{a},\bar{b}\in D\;{\bar{a}-}_{D}\bar{b}=\left[a^{-}-b^{-},{\;a}^{+}-b^{+}\right]$$
- Directed division:
  (7) $$\bar{a}{/}_{D}\bar{b}=\left\{\begin{array}{c} \left[a^{-\sigma \left(\bar{b}\right)}/b^{-\sigma \left(\bar{a}\right)},a^{\sigma \left(\bar{b}\right)}/b^{\sigma \left(\bar{a}\right)}\right],\;\;\;\;\;\;\;\;\bar{a},\bar{b}\in D\backslash Z\\ \left[a^{-\sigma \left(\bar{b}\right)}/b^{\sigma \left(\bar{b}\right)},a^{\sigma \left(\bar{b}\right)}/b^{\sigma \left(b\right)}\right],\;\;\;\;\;\;\;\;\;\;\bar{a}\in Z,\bar{b}\in D\backslash Z \end{array}\right.$$

where *σ* denotes the sign functional, as follows:


(8) $$\forall \bar{a}\in D\backslash Z\;\;\;\;\sigma \left(\bar{a}\right)=\left\{\begin{array}{c} +,\;\;\;\;\;\;\;{if\;a}^{-},a^{+}>0\\ -,\;\;\;\;\;\;\;{if\;a}^{-},a^{+}<0 \end{array}\;\right.$$

Thus, one obtains ${\bar{a}-\;}_{D}\bar{a}=\bar{0}\;$ and ${\bar{a}/}_{D}\bar{a}=\bar{1}$. These definitions make it possible to perform subtraction and division on interval numbers more effectively, while considerably limiting the undesired growth of interval widths [67]. Moreover, the difference in interval numbers appearing in the objective function can assume a value close to, or even equal to, zero. This property is particularly beneficial in optimisation problems, where minimising the width of such differences is often required.

In ref. [36], the authors present the application of fuzzy numbers representing inaccuracies in the computational homogenization of composite structures with imprecise parameters. In this case, fuzzy numbers are represented by their α-cuts, which allows for the use of directed interval arithmetic.

### 2.2. Computational Interval Homogenisation

Multiscale modelling allows for the modelling of structures at different length scales. Computational homogenisation is based on the concept of a representative volume element (RVE) or a unit cell for periodic structures, e.g., cellular auxetic materials. In the present paper, the unit cell representation is considered. The unit cell represents the repeating unit of geometry of a periodic structure. In the homogenization, usually the following criteria are assumed [68]:

- The Hill–Mandel condition, which states that the microscopic average energy density within a unit cell is equal to the macroscopic energy density at the corresponding point in the macrostructure. Typically, volume averages of strains and stresses can be replaced by boundary integrals [69], as follows:
  (9) $$\begin{array}{c} \begin{array}{c} \langle \sigma_{ij}\varepsilon_{ij}\rangle =\langle \sigma_{ij}\rangle \langle \varepsilon_{ij}\rangle \\ \langle \sigma_{ij}\rangle =\frac{1}{\left|V\right|}\int_{V}\sigma_{ij}dV=\frac{1}{\left|V\right|}\int_{\Gamma }t_{i}x_{j}d\Gamma \end{array}\\ \langle \varepsilon_{ij}\rangle =\frac{1}{2\left|V\right|}\int_{\Gamma }\left(u_{i}n_{j}+u_{j}n_{i}\right)d\Gamma \end{array}$$
  where $\sigma_{ij}$—micro stress tensor, $\varepsilon_{ij}$—micro strain tensor, 〈∙〉—average quantity, V—the unit cell volume, *Γ*—the external boundary of unit cell, *t_i_*—traction force component, *x_j_*—coordinates, *u_i_*—displacement component, *n_j_*—unit normal vector to the boundary.
- The boundary conditions that satisfy the Hill–Mandel condition. In the present article, periodic boundary conditions are employed:
  (10) $$\begin{array}{c} u_{i}^{pos}-u_{i}^{neg}=\langle \varepsilon_{ij}\rangle \cdot \left(x_{i}^{pos}-x_{i}^{neg}\right)\\ {\;t}_{i}^{pos}={-t}_{i}^{neg},\;\;\;\;\;\;\;\;\forall x\in \Gamma :n_{i}^{pos}={-n}_{i}^{neg} \end{array}$$
  where $u_{i}^{pos}$, $u_{i}^{neg}$—displacements of the corresponding points at the opposite unit cell boundaries, $x_{i}^{pos}$, $x_{i}^{neg}$—locations of the corresponding points at the opposite unit cell boundaries, $t_{i}^{pos}$, $t_{i}^{neg}$—tractions on the corresponding points at the opposite unit cell boundaries, $n_{i}^{pos}$, $n_{i}^{neg}$—normal vectors at the opposite unit cell boundaries.

In the case of nonlinear materials, the microscopic stress σ(x,t) at given time *t* of the RVE/unit cell quasi-static deformation process is related to microscopic strain ε(x,t) by a nonlinear operator $G\left(\cdot \right)$ [69], as follows:

(11) $$\sigma \left(x,t\right)=G\left[\varepsilon \left(x,t\right),\;\varepsilon^{p}\left(x,t\right)\right]$$

with plastic strains within the RVE/unit cell $\varepsilon^{p}\left(x,t\right)$, satisfying the following:

(12) $$\langle \varepsilon (x,t)\rangle =\hat{\varepsilon }(t)$$

where $\hat{\varepsilon }(t)$ is a macroscopic strain. The procedure comprises the following steps. Given $\left(x,t\right),\varepsilon^{p}\left(x,t\right):$

1. Prescribe the boundary conditions on Γ.
2. Solve the nonlinear BVP related to RVE/unit cell by FEM.
3. Average the stress in the RVE/unit cell in equilibrium to obtain corresponding $\langle \sigma (x,t)\rangle =\hat{\sigma }\left(t\right)$, where $\hat{\sigma }\left(t\right)$ is a macroscopic stress.

Thus, the constitutive law $\hat{\sigma }\left(t\right)$-$\hat{\varepsilon }(t)$ is determined numerically. The problem is solved for *T* points in time $t_{l}\;(l=1,2,\ldots ,T)$ to obtain discrete points of the constitutive law ${\hat{\sigma }}^{l}$-${\hat{\varepsilon }}^{l}$, with increasing ${\hat{\varepsilon }}^{l}$. A more practical method for calculating the average stress in FEM analysis, which is applied here, is based on summing the forces at the control nodes on the external boundary of the RVE/unit cell. The transition from the volume integral based on stresses to the integral that involves boundary tractions, and ultimately the sum of external forces relevant in FEM analysis, is discussed in detail in [35].

An application of FEM to solve the BVP is time-consuming, which is especially inconvenient in the identification problems which require multiple calculations of the objective function(s) value. To reduce computational costs, metamodels in the form of response surfaces (RSs)—here, ANNs—are employed [70]. The way in which ANNs work is inspired by the way in which biological neurons form the human brain and nervous system [71]. An artificial neuron receives multiple input signals, each scaled by an associated weight coefficient, and generates a single scalar output signal. In a standard feedforward architecture, neurons are arranged in successive interconnected layers: an input layer, one or more hidden layers (if required), and an output layer. The output *y_i_* for an *i*-th neuron of the ANN is determined by the following:

(13) $$y_{i}=\omega \left(\sum_{j=0}^{N}w_{ij}z_{j}\right)=\omega \left(\sum_{j=1}^{N}w_{ij}z_{j}+B\right)$$

where $y_{i}$—an output value the *i*-th neuron, ω—an activation function, $w_{ij}$—a weighting coefficient for *j*-th input value, $z_{j}$—a *j*-th input value of the *i*-th neuron, $B=w_{i0}z_{0}$—neuron bias.

The choice of activation function depends on the nature of the problem. In this study, a sigmoid function is utilised, as follows:

(14) $$\omega \left(x\right)=\frac{1}{1+e^{-\psi z}}$$

where *ψ*—a parameter that defines the steepness of the function’s slope.

To account for the effects of uncertainties, the neural network is reformulated into a set of algebraic equations (Equation (13)), that describe the output parameter values, i.e., a response surface representation. In this form, the response surface is constructed using the fixed weight coefficients and bias terms ($w_{ij}$, B) of the neural network, combined with variable input parameters ($z_{j}$). To enable a direct propagation of input uncertainties to the output parameters, interval arithmetic is applied in the response surface calculations. Consequently, when the input parameters are expressed as interval numbers, the uncertainties are consistently incorporated at every stage of mathematical operations, as follows:

(15) $${\bar{y}}_{k}=\omega \left(\sum_{k=1}w_{k}{\bar{z}}_{k}+B\right)$$

where ${\bar{y}}_{k}$—an interval output value, $w_{k}$—a weighting coefficient, ${\bar{z}}_{k}$—an interval input value. As the calculation of the activation function ω requires the exponential function utilisation, the interval exponential operation is given as follows:

(16) $$e^{\bar{a}}=\left[e^{a-},\;e^{a+}\;\right]$$

Additionally, the extension of the interval operations for degenerate intervals of the type $A=\left[a,a\right]$ was applied [65].

**Figure 1:**
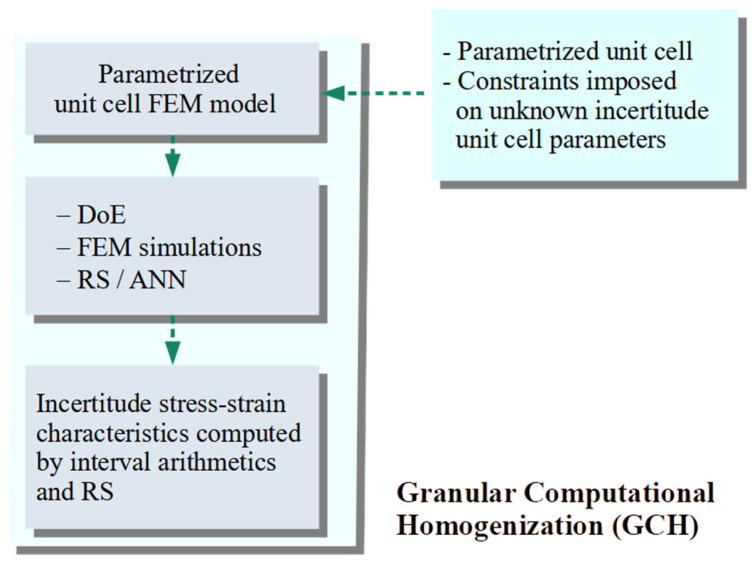
The GCH data flow.

In the present paper, a Granular Computational Homogenisation (GCH) procedure is proposed. GCH is predicated on the representation of the microstructure of the given material, response surfaces, and directed interval arithmetic. Boundary-value problems at the microscale are solved using ANSYS 2023R2 finite element method software. The GCH scheme is presented in Figure 1. The procedure starts with the creation of a geometric model of the structure (unit cell) based on the microstructure of the decision on uncertain parameters. The determination of geometry and material properties as either certain or uncertain results in the number of input parameters that are taken into account. The model is transferred to FEM ANSYS 2023R2 software to create a numerical model of the microstructure, including its constitutive relationships. In the next step, an RS is created with the use of the Design of Experiment (DoE). The RS is generated from unit cells computed using conventional non-interval numerical homogenisation. The Levenberg–Marquardt algorithm implemented in MATLAB R2023a software [72] is applied as a learning algorithm for the ANN. The Maximin Distance algorithm [73], which belongs to the class of Optimal Space Filling techniques, is employed as a DoE. The quality of the RS is determined by the mean square error (MSE) and the coefficient of determination (*R*^2^) metrics.

The metamodel is expressed as a system of algebraic relations mapping the input parameters onto their corresponding outputs. In the next stage, these relations are reformulated using directed interval arithmetic to yield the interval RS. Subsequent interval computations generate output parameters in the form of interval-valued stiffness matrix coefficients for linear materials and interval stress–strain relations for nonlinear materials.

### 2.3. Computational Interval Identification

The computational interval identification is the search for microscale property ranges of the model that result in specific macroscale material behaviour. This can be considered as a constrained optimisation problem, described as follows:

(17) $$\left\{\begin{array}{c} minimise\;\;f_{g}\left(\bar{p}\right)\\ subject\;to:\;b_{i}^{L}\leq p_{i}^{-,+}\leq b_{i}^{U},\;\;\;\;\;\;\;\;i=1,\;2,\ldots ,\;n \end{array}\;\right.$$

where $\bar{p}$—the vector of design variables, $f_{g}\left(\bar{p}\right)$—the objective function, and n—the number of design variables.

The resulting interval stiffness coefficients serve as the basis for constructing the objective function within the optimisation algorithm. Their purpose is to evaluate how well they align with the predefined uncertain material property values. The discrepancy, or distance, between two interval numbers is quantified using the following norm:

(18) $$D\left(\left[a^{-},a^{+}\right],\;\left[b^{-},b^{+}\right]\right)=\sqrt{{\left(a^{-}-b^{-}\right)}^{2}+{\left(a^{+}-b^{+}\right)}^{2}}$$

In the context of multi-objective optimisation of nonlinear materials, the *k*-th objective function is described by the following:

(19) $$f_{k}\left(\bar{p},{\hat{\varepsilon }}^{l}\right)=\underset{l}{max}\left\{D\left[{\bar{\hat{\sigma }}}_{ij}^{l}\left(\bar{p},{\hat{\varepsilon }}_{ij}^{l}\right),{\bar{\hat{\sigma }}}_{ij}^{*l}\left({\hat{\varepsilon }}_{ij}^{l}\right)\right]\right\}\;\;\to min,\;\;\;\;\;\;\;l=1,\;\ldots ,T$$

where ${\bar{\hat{\sigma }}}^{l}\left(\bar{p},{\hat{\varepsilon }}_{ij}^{l}\right)$—an interval stress function calculated from RS, ${\bar{\hat{\sigma }}}_{ij}^{*l}\left({\hat{\varepsilon }}_{ij}^{l}\right)$—assumed output interval stress, *T*—the number of strain samples.

In the present paper, a Granular Computational Inverse Design (GCID) methodology is proposed [74]. This methodology introduces an additional objective related to the width of the input parameter ranges. To provide an equivalent treatment of all parameters, a width scaling is applied, as follows:

(20) $$w^{\prime }\left({\bar{p}}_{i}\right)=\frac{w\left({\bar{p}}_{i}\right)}{\overset{ˇ}{p}}$$

where $w\left({\bar{p}}_{i}\right)$—a current width of the *i*-th interval parameter, $\overset{ˇ}{p}$—the midpoint of the *i*-th interval parameter.

Then, the objective function describing the minimum width takes the following form:

(21) $$f_{k}\left(\bar{p},{\hat{\varepsilon }}^{l}\right)=\underset{p_{i}}{\min}\;w\prime \left({\bar{p}}_{i}\right)\;\to max,\;\;\;\;\;\;\;i=1,\ldots ,n$$

where *n*—the number of input parameters.

**Figure 2:**
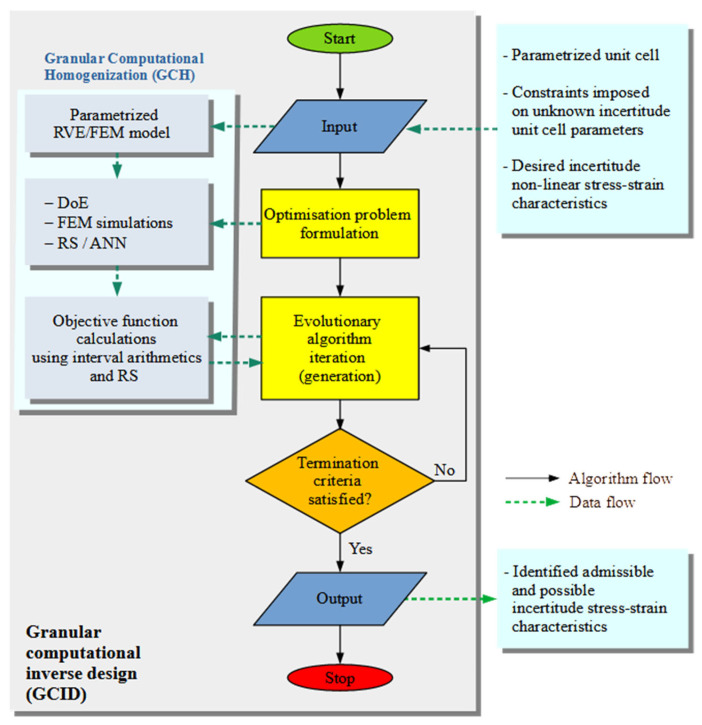
The GCID scheme.

The GCID methodology scheme is presented in Figure 2. The process begins with the formulation of the optimisation problem by defining the following parameters: the number of design variables, the number of stiffness coefficients to be analysed, the constraints applied to these variables, and the set of optimisation criteria, determining the single- or multi-objective approach. The next stage uses the GCH methodology of the previous section to determine the output interval stiffness coefficients from the interval variables. The values of the objective functions are calculated from the output values. This sequence continues until the termination criterion specified by the optimisation algorithm is met.

The GCID methodology employs a standard optimisation algorithm (formulated in classical arithmetic) to address problems with interval uncertainty by transforming the design variables. This transformation is achieved by introducing two input variables, ($a^{-}$ and $a^{+}$), corresponding to the bounds of each interval parameter. The proper interval condition is enforced by introducing linear constraints on the deterministic input parameters, as follows:

(22) $$A_{l}\cdot p\leq b_{l}$$

where p—a 2n×1 vector of input parameters, $b_{l}$ is a n×1 zero vector, $A_{l}$—an n×2n constraint matrix, as follows:

(23) $$A_{l}=\left[\begin{array}{ccccccc} 1 & -1 & 0 & 0 & \ldots & 0 & 0\\ 0 & 0 & 1 & -1 & \ldots & 0 & 0\\ \vdots & \vdots & \vdots & \vdots & \ddots & \vdots & \vdots \\ 0 & 0 & 0 & 0 & \ldots & 1 & -1 \end{array}\right]$$

This paper adapts the Pareto approach to multi-objective optimisation. A multi-objective evolutionary algorithm built into MATLAB software [75] is used to perform the optimisation. The result is a Pareto front of non-dominated solutions, which are equal solutions of the optimisation task. To evaluate the quality of obtained Pareto front, the hypervolume indicator *I_H_* is calculated [76].

In engineering practice, the Pareto approach to multi-objective optimisation requires a second step to select one (or more) solutions, which usually requires a decision maker. This step is beyond the scope of the present study.

## 3. Numerical Examples

### 3.1. Computational Interval Homogenisation of Auxetic Structure

#### 3.1.1. Model Establishment for the Interval Homogenisation

**Figure 3:**
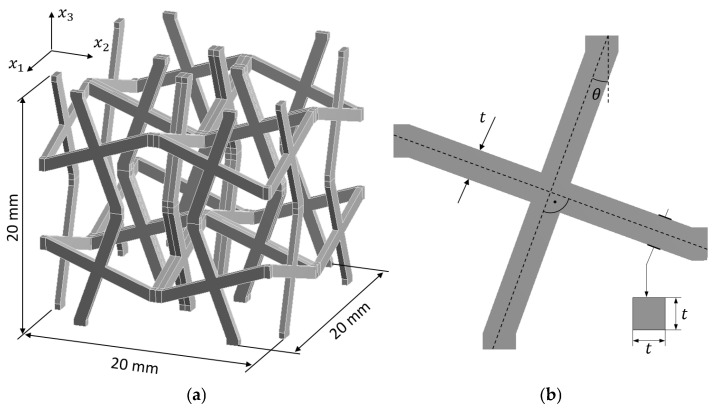
Unit cell for the cross chiral structure: (**a**) dimensions and coordinate system and (**b**) 2D representation.

An auxetic cellular material made of PA12 polyamide with cross chiral microstructure (Figure 3a) is considered [77]. As the material constants for homogeneous material are usually determined through experimental methods, some of them are approximated with a certain degree of uncertainty. The remaining properties are assumed to be constant and certain. The elastic–plastic properties are described in terms of the bilinear isotropic hardening rule and are represented by interval numbers. Therefore, the Young’s modulus (*E*) and yield strength (*R_e_*) are determined to be 850 MPa ± 2% and 30 MPa ± 4%, respectively. The specific properties are as follows: Poisson’s ratio *ν* = 0.4 and tangent modulus *E*_*T*_ = 87 MPa. Furthermore, geometric inaccuracies resulting from the manufacturing process are also taken into account by introducing the following two additional uncertain geometry parameters: structure thickness *t* = 1 mm ± 5% and tilt angle *θ* = 20° ± 2% (Figure 3b).

The aim is to determine the possible ranges of equivalent macroscopic nonlinear material properties caused by loading normal strain in two directions (*x*_1_ and *x*_3_), depending on the assumed uncertainties. In order to solve this problem, computational homogenisation based on a representation of the material microstructure and an RS in the form of an ANN are used.

The microstructure of the material is represented by a unit cell whose regular geometry makes the material orthotropic with the following two perpendicular directions: *x*_1_ and *x*_2_. Consequently, the following relationships hold: $\sigma_{11}=\sigma_{22}$, $\sigma_{13}=\sigma_{23}$, and $\sigma_{44}=\sigma_{55}$. As only a nonlinear response to normal strain is important, only two nonlinear normal strain tests are required, as follows: one with non-zero $\varepsilon_{11}$ (other $\varepsilon_{ij}=0$) to evaluate $\sigma_{11}$, $\sigma_{12}$, and $\sigma_{13}$, and one with non-zero $\varepsilon_{33}\;$(other $\varepsilon_{ij}=0$) to evaluate $\sigma_{31}$ and $\sigma_{33}$. The model’s behaviour under high deformation conditions is ensured through the consideration of nonlinear geometric effects during the simulation.

**Figure 4:**
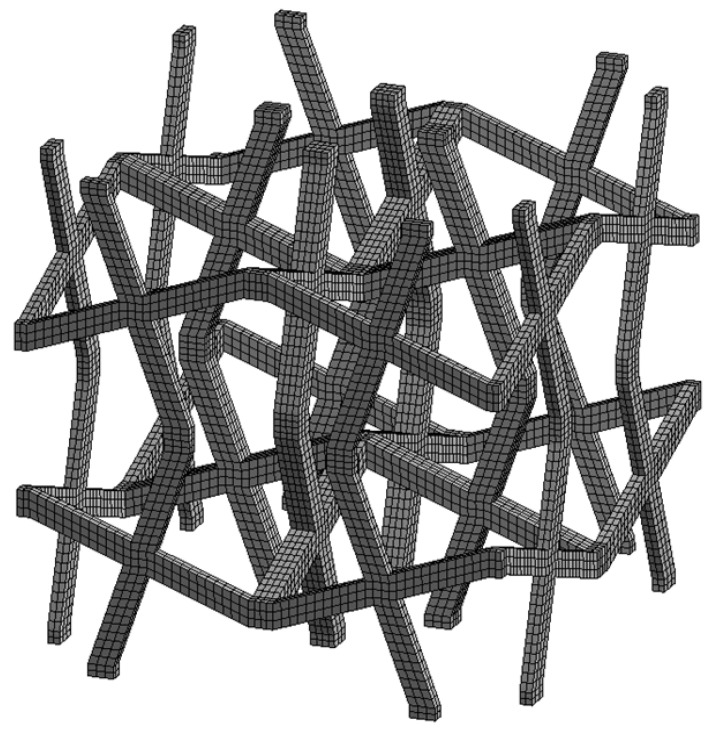
Typical cross chiral structure modelled in the ANSYS software. Parameters: Hex20 elements, periodic boundary conditions, bilinear elastic-plastic model of PA12 polyamide material with isotropic hardening rule, geometry parameters: *t* = 1 mm, *θ* = 20°.

The unit cell is divided into 10,624 hexahedral finite elements (Figure 4) with quadratic shape functions (Hex20 in ANSYS), resulting in 184,677 degrees of freedom (DoF).

The model results from a preliminary convergence check that involves various mesh densities and shape functions, specifically linear and quadratic options. We consider the following factors: the maximum equivalent stress at the microscopic level, the maximum equivalent plastic strain, the macroscopic stress components, and the computation time. Our criterion for convergence is that the macroscopic stress components fall within a tolerance of 1%, alongside reasonable computation time. This level of tolerance should not significantly impact the uncertainties associated with the resulting macroscopic stresses. It is important to note that to accurately model the strength of the structure, which is influenced by local stress and strain fields, finer models or/and enrichment finite element strategies [78] should be employed. The relative discrepancy between the theoretical and discretised geometric volumes depends on the geometric parameters, with a maximum observed value of 6.329 × 10^−8^%. Therefore, it is assumed not to affect the uncertain geometrical parameters.

#### 3.1.2. Numerical Results of the Interval Homogenisation

The creation of the RS was preceded by DoE with 80 design points that discretised the input parameters’ space. During the FEM analysis, the stress response was recorded for 25 incrementing strain load values $\varepsilon_{11},\;\varepsilon_{33}\in \langle 0,\;0.1\rangle$. The strain load value $\varepsilon_{ij}\;$ was employed as an additional input parameter. The calculation time for each design point was approximately 25 min, resulting in an overall calculation time for all design points of approximately 33 h.

**Figure 5:**
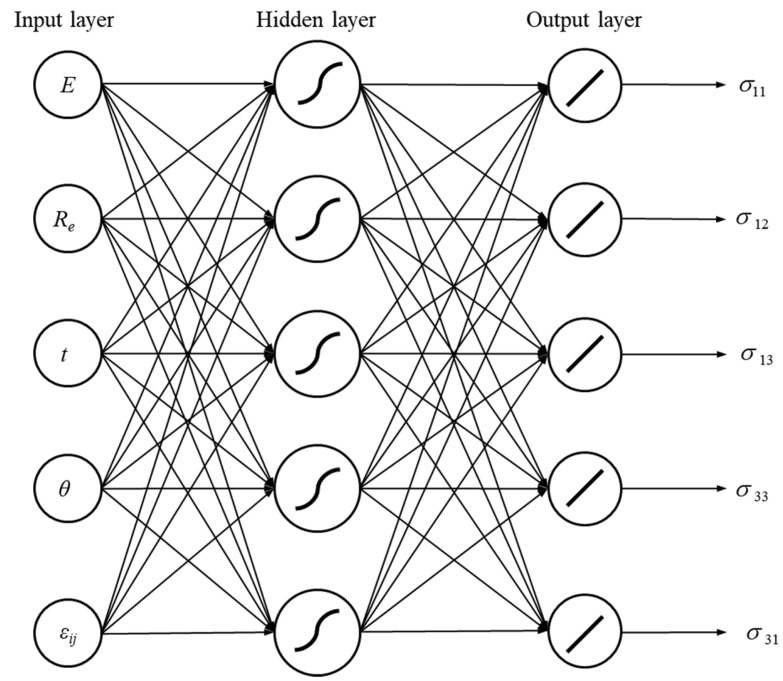
ANN structure.

An ANN was then created with the structure (5-5-5), as shown in Figure 5. The thick lines inside the neurons represent, respectively: a sigmoidal activation function (hidden layer) and a linear activation function (output layer). The parameter *ψ* of the sigmoid activation function was assumed to be equal to two. In total, 70% of the calculated set of DoE parameters was used to train the ANN, while the remaining part was used to test the network. The learning algorithm converged after 692 learning epochs, with an MSE of 4.497 × 10^−3^ MPa^2^ and an R^2^ of 0.999, indicating the high quality of the RS.

**Figure 6:**
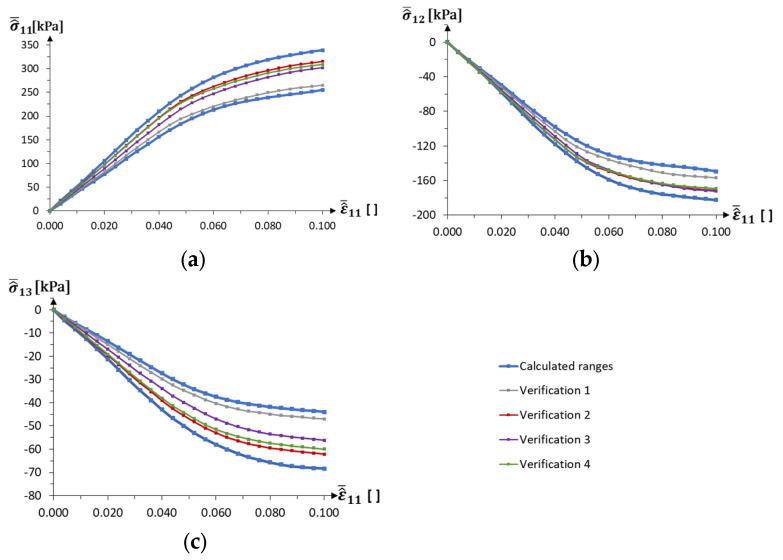

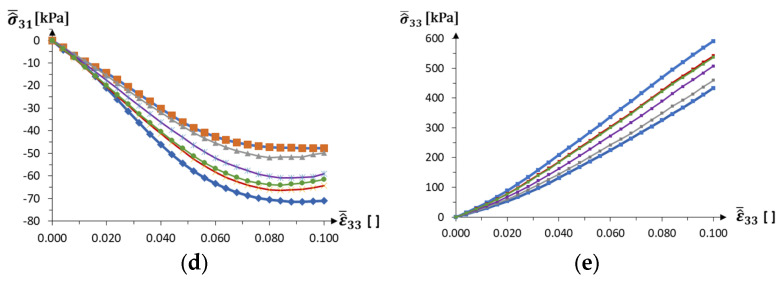
Nonlinear material stress–strain curves for auxetic material, with results for random parameter sets (**a**–**c**) For $\varepsilon_{11}$ ≠ 0, (**d**,**e**) For $\varepsilon_{33}$ ≠ 0, (remaining $\varepsilon_{ij}$ = 0).

Uncertainty is analysed using the interval values for four parameters, resulting in interval equations. The strain parameter is an additional certain variable. The blue curves in Figure 6 show the two-dimensional ${\bar{\hat{\sigma }}}_{ij}-{\bar{\hat{\varepsilon }}}_{11}$ and ${\bar{\hat{\sigma }}}_{ij}-{\bar{\hat{\varepsilon }}}_{33}\;$ interval relationships. The ranges obtained are verified by calculating four sets of random parameters using numerical model calculations without applying neural networks and interval arithmetic. These curves are presented in Figure 6, using additional colours. Negative values of ${\bar{\hat{\sigma }}}_{13}$ and ${\bar{\hat{\sigma }}}_{31}$ prove that the structure is auxetic.

### 3.2. Computational Interval Identification of Auxetic Structure

#### 3.2.1. Model Establishment for the Interval Identification

**Figure 7:**
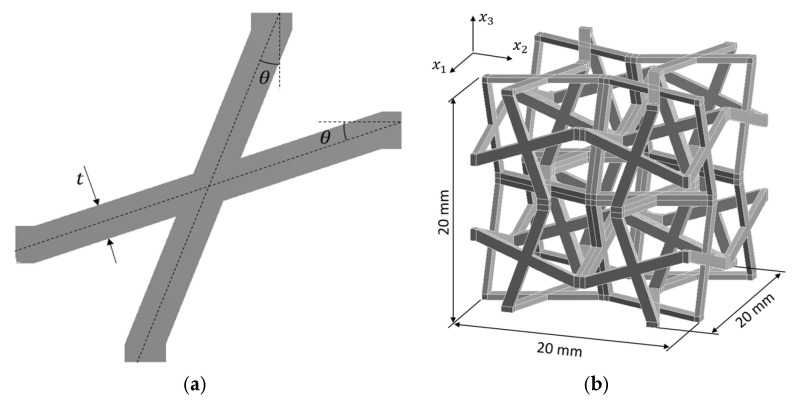
Unit cell for the reinforced cross chiral structure: (**a**) 2D representation of additional reinforcement and (**b**) dimensions and coordinate system.

An auxetic cellular material made of PA12 polyamide with a modified cross chiral microstructure (Figure 7) is considered [77]. The modification consists of introducing supplementary structural reinforcement within the material illustrated in the preceding numerical example.

**Figure 8:**
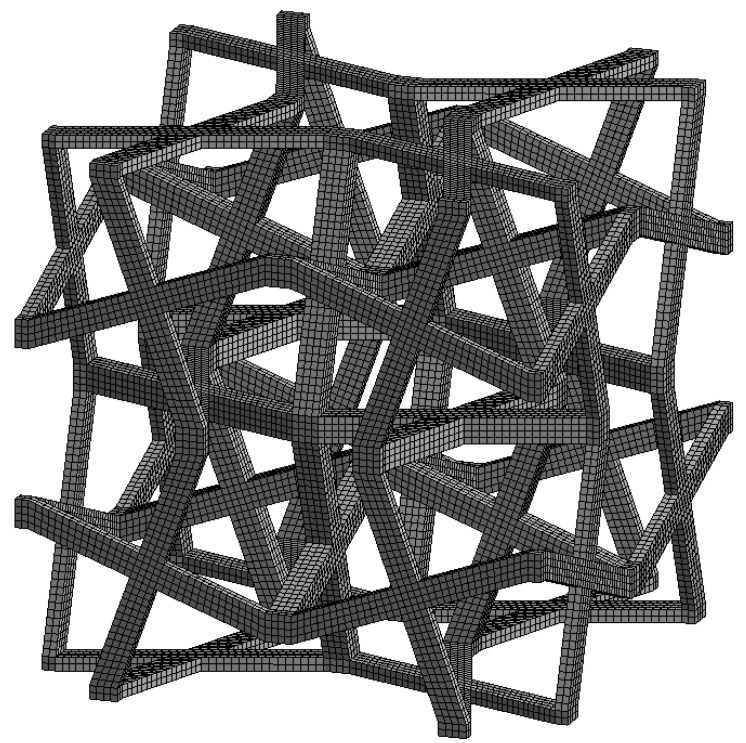
Typical modified cross chiral structure modelled in the ANSYS software. Parameters: Hex20 elements, periodic boundary conditions, bilinear elastic-plastic model of PA12 polyamide material with isotropic hardening rule, geometry parameters: *t* = 1 mm, *θ* = 20°.

The unit cell was divided into 27,648 20-node hexahedral finite elements with quadratic shape functions (Figure 8), resulting in 457,197 DoFs. The purpose of the optimisation process was to identify the possible ranges of microscale input parameters, enabling the derivation of equivalent nonlinear macroscopic material properties associated with normal strain loading in the *x*_1_ and *x*_3_ directions. Computational homogenisation based on material microstructure representation, RS in the form of an ANN, interval numbers, and multi-objective EA were used to solve the identification task.

Three contradictory objectives are simultaneously considered. The first two objectives are to obtain certain values for $\sigma_{11}$ and $\sigma_{33}$, which should be described by a given interval function. The third objective concerns the width of the ranges of the design variables. The thickness, tilt angle, elastic modulus, and yield strength of the structure material are used as interval design variables, as follows: ${\bar{p}}_{1}=\bar{t}\;\left[mm\right],{\bar{p}}_{2}=\bar{\theta \;}\left[{}^{\circ}\right],{\bar{p}}_{3}=\bar{E}\;\left[MPa\right],{\;\bar{p}}_{4}={\bar{R}}_{e}\;\left[MPa\right]$.

The optimisation problem is described by the following:

(24) $$\left\{\begin{array}{c} \begin{array}{c} minimise\;f_{1}\left(\bar{p}\right)\\ minimise\;f_{2}\left(\bar{p}\right)\\ maximise\;f_{3}\left(\bar{p}\right) \end{array}\\ subject\;to:\;\left\{\begin{array}{c} f_{1}\left(\bar{p},{\hat{\varepsilon }}^{l}\right)=\underset{l}{max}\left\{D\left[{\bar{\hat{\sigma }}}_{11}^{l},{\bar{\hat{\sigma }}}_{11}^{*l}\left({\hat{\varepsilon }}_{11}^{l}\right)\right]\right\},\;\;\;\;\;\;\;\;l=1,\;\ldots ,15\\ f_{2}\left(\bar{p}{,\hat{\varepsilon }}^{l}\right)=\underset{\varepsilon_{i}}{max\;}\left\{D\left[{\bar{\hat{\sigma }}}_{33}^{l},{\bar{\hat{\sigma }}}_{33}^{*l}\left({\hat{\varepsilon }}_{33}^{l}\right)\right]\right\},\;\;\;\;\;\;l=1,\ldots ,15\\ f_{3}\left(\bar{p}\right)=\underset{p_{j}}{\min}\;w^{\prime }\left({\bar{p}}_{j}\right),\;\;\;\;\;\;\;\;\;\;\;\;\;\;\;\;j=1,\ldots ,4\\ b_{Li}\leq p_{i}^{-,+}\leq b_{Ui},\;\;\;\;\;\;\;\;\;\;\;\;j=1,\ldots ,4\\ {\bar{b}}_{LU}=\left[\begin{array}{rr} 0.8 & 1.4\\ 15.0 & 25.0\\ 700.0 & 1000.0\\ 20.0 & 40.0 \end{array}\right] \end{array}\right. \end{array}\right.$$

where $\bar{p}={\left[{\bar{p}}_{1},{\bar{p}}_{2},{\bar{p}}_{3},{\bar{p}}_{4}\right]}^{T}$ is the vector of design variables.

The interval functions that describe the behaviour of the desired stiffness coefficients are initially assumed. Their values are collected in Table 1.

#### 3.2.2. Numerical Results of the Interval Identification

The procedure to generate an RS in the form of ANN is identical to the previous numerical example. The structure of the ANN is the same as that presented in Figure 5. DoE using the Optimal Space Filling algorithm is used to generate 100 design points. During the analysis, the stress response is stored for 15 increasing strain load values, as follows: $\varepsilon_{11},\;\varepsilon_{33}\in \langle 0,\;0.125\rangle$. The elastic–plastic properties of the material for the RS generation are *E* = 850 MPa ± 16.7%, ν = 0.4, *R_e_* = 30 MPa ± 33.3%, and *E*_*T*_ = 87 MPa. The assumed ranges of geometrical properties are *t* = 1 mm ± 60%, and *θ* = 20° ± 20%.

To limit the search area for the Pareto front, the following nonlinear constraints are introduced: $f_{1}\left(\bar{p}\right)<0.1$ MPa, $f_{2}\left(\bar{p}\right)<0.1$ MPa and $f_{3}\left(\bar{p}\right)<0.1$. The multi-objective evolutionary algorithm [75] us used to perform the optimisation. The parameters of the algorithm are as follows.

- Number of individuals *n_i_* = 200;
- Scatter crossover probability *c_s_* = 0.8;
- Number of generations *n_g_* = 1000;
- Number of stall generations *ns* = 50;
- Pareto fraction set *P_f_* = 0.5.

**Figure 9:**
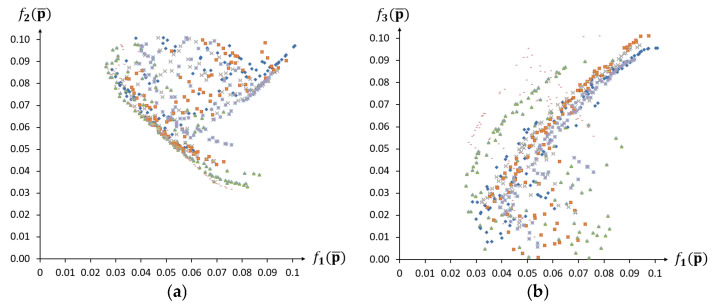

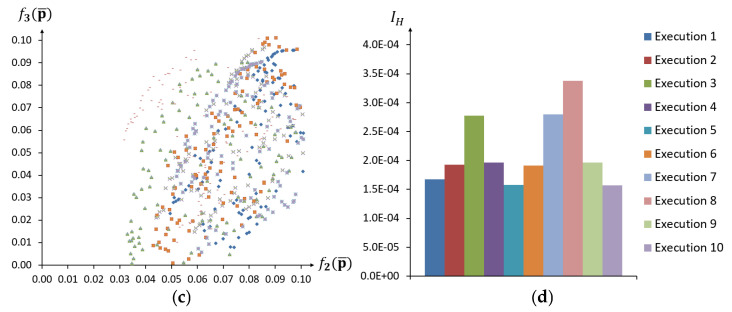
Pareto fronts and hypervolume indicator values for 10 independent runs of the algorithm: (**a**) $f_{1}\left(\bar{p}\right)-f_{2}\left(\bar{p}\right)$, (**b**) $f_{1}\left(\bar{p}\right)-f_{3}\left(\bar{p}\right)$, (**c**) $f_{2}\left(\bar{p}\right)-f_{3}\left(\bar{p}\right)$, and (**d**) $I_{H}$.

The results of the multi-objective optimisation for 10 independent runs of the algorithm are presented in the form of Pareto fronts and hypervolume indicator values in Figure 9.

**Figure 10:**
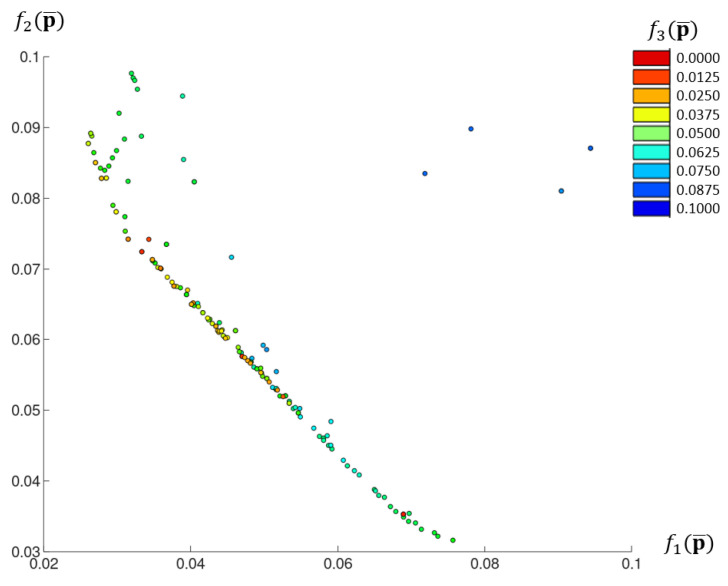
Aggregated Pareto front for all runs of the evolutionary algorithm.

Figure 10 shows an aggregated Pareto front from combining the solutions from different algorithm executions.

The results for the minimum values of $f_{1}\left(\bar{p}\right)$ and $f_{2}\left(\bar{p}\right)$, the maximum value of $f_{3}\left(\bar{p}\right)$, and the closest to an ideal point (0, 0, 0.1), together with the values of the resulting design parameters, are presented in Table 2.

**Figure 11:**
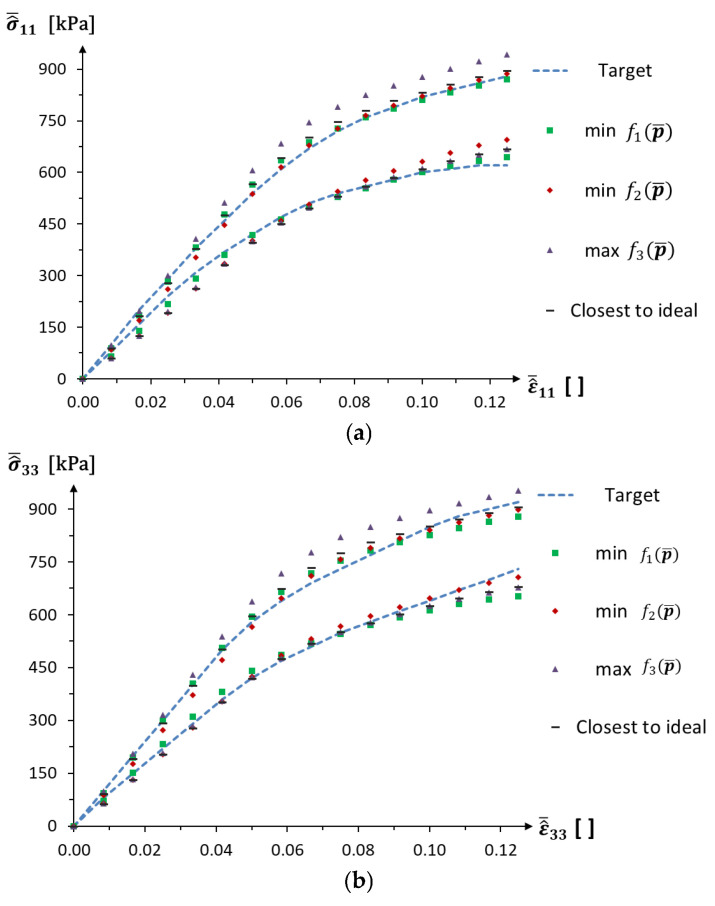
Optimised nonlinear stress–strain curves: (**a**) ${\bar{\hat{\sigma }}}_{11}$-${\bar{\hat{\varepsilon }}}_{11}$ and (**b**) ${\bar{\hat{\sigma }}}_{33}-{\bar{\hat{\varepsilon }}}_{33}$.

The nonlinear stress–strain curves for individuals from chosen algorithm executions are presented in Figure 11.

## 4. Discussion

Both numerical examples consider auxetic materials, taking into account the nonlinear material properties and deformations of the structure during loading. The results of the computational interval homogenisation show the nonlinear behaviour of the unit cell model during normal stress loading in the *x*_1_ and *x*_3_ directions (Figure 6). In addition, it can be observed that the width of the set described by the interval number increases with strain, which means that the uncertainty in material behaviour also increases for higher strain values. Verification of the results obtained by calculating four sets of random parameters for the numerical model without neural networks and interval arithmetic shows that the results do not exceed the ranges calculated using the interval approach.

The results of the identification demonstrate that, in the context of conflicting objective functions, it is not possible to obtain a solution that precisely satisfies both stress–strain curves under consideration. The results of running the algorithm multiple times also indicate that equivalent minimal values for the objective functions $f_{1}\left(\bar{p}\right)$ and $f_{2}\left(\bar{p}\right)$ are achieved for differing degrees of uncertainty in the design parameters. For example, the Pareto fronts of the third and eighth runs of the algorithm nearly coincide on the $f_{1}\left(\bar{p}\right)$-$f_{2}\left(\bar{p}\right)\;$ plane (Figure 9a) but are in different parts of the graph for the other projections (Figure 9b,c). Thus, it can be concluded that, given the constraints imposed by the objective function $f_{3}\left(\bar{p}\right)$, an increase in the uncertainty of the design parameters does not necessarily lead to drift beyond the assumed stress–strain curves. Moreover, the results from the aggregated Pareto front show that increasing the $f_{3}\left(\bar{p}\right)\;$ constraint value can lead to an increase in the uncertainty of the design parameters, without significantly worsening the first two objective functions. The results for the multi-objective algorithm executions allow for the selection of optimal solutions from the Pareto front, which exhibit good agreement with the required behaviour and also satisfy the optimisation constraints.

## 5. Conclusions

In the present paper, a novel framework is proposed that effectively handles interval uncertainties in the macroscopic stress–strain response of cellular auxetic structures. The proposed framework requires testing the finite element model’s quality and assessing the convergence of the quantities of interest within a tolerance that is negligible compared to the uncertainties being considered.

The numerical examples provided primarily focus on the uniaxial strain nonlinear response. However, the proposed framework is applicable to both shear and full orthotropic responses of the structure being analysed. It is important to note that there are no methodological restrictions on how the structure can be loaded within this framework. Obviously, additional loading tests will necessitate more computational effort.

The examples presented demonstrate the effectiveness of the GCH and GCID methodologies in solving computational homogenisation problems, as well as optimisation and identification in multiscale problems. The required macroscopic properties of the material are obtained with satisfactory accuracy and in a time-efficient manner. The use of an RS and directed interval arithmetic facilitates a substantial reduction in computational effort. The authors intend to extend the presented approach to similar issues related to the uncertainties represented by other information granularity models, especially fuzzy numbers.

## Figures and Tables

**Table 1:** Values of desired stress intervals for optimisation of nonlinear auxetic material.

${\hat{\varepsilon }}_{11}^{l},{\hat{\varepsilon }}_{33}^{l}[]$		0.0083	0.0167	0.025	0.0333	0.0417	0.050	0.0583	0.0667
${\bar{\hat{\sigma }}}_{11}^{*l}\left({\hat{\varepsilon }}_{11}^{l}\right)$ [kPa]	min	80	160	240	310	370	420	470	510
	max	100	200	290	380	460	540	610	670
${\bar{\hat{\sigma }}}_{33}^{*l}\left({\hat{\varepsilon }}_{33}^{l}\right)$ [kPa]	min	80	150	220	290	360	420	470	510
	max	100	200	300	400	500	580	640	690
${\hat{\varepsilon }}_{11}^{l},{\hat{\varepsilon }}_{33}^{l}[]$		**0.075**	**0.0833**	**0.0917**	**0.1000**	**0.1083**	**0.1167**	**0.125**	
${\bar{\hat{\sigma }}}_{11}^{*l}\left({\hat{\varepsilon }}_{11}^{l}\right)$ [kPa]	min	540	560	580	600	610	620	620	
	max	720	760	790	820	840	860	880	
${\bar{\hat{\sigma }}}_{33}^{*l}\left({\hat{\varepsilon }}_{33}^{l}\right)$ [kPa]	min	550	580	610	640	670	700	730	
	max	730	770	810	850	880	900	920	

**Table 2:** Optimisation results for multi-objective optimisation of reinforced auxetic material.

Pareto Point	$\min\;f_{1}\left(\bar{p}\right)$	$\min\;f_{2}\left(\bar{p}\right)$	$\max\;f_{3}\left(\bar{p}\right)$	Closest to Ideal
${\bar{p}}_{1}$ = t [mm]	[1.0788, 1.1232]	[1.0238, 1.0822]	[1.0145, 1.1315]	[1.0112, 1.1028]
${\bar{p}}_{2}$ = θ [^o^]	[22.2422, 22.9978]	[19.9566, 22.1034]	[19.2005, 21.2595]	[19.4915, 21.3485]
${\bar{p}}_{3}$ = E [MPa]	[745.4873, 875.9127]	[750.6961, 828.3039]	[751.5213, 831.6787]	[751.0145, 832.3855]
${\bar{p}}_{4}$$\;=\;R_{e}$ [MPa]	[26.2683, 33.9317]	[33.3576, 37.2024]	[31.9203, 35.2797]	[32.3513, 35.6487]
$f_{1}\left(\bar{p}\right)$	2.6072 × 10^−2^	7.5659 × 10^−2^	7.8118 × 10^−2^	5.1137 × 10^−2^
$f_{2}\left(\bar{p}\right)$	8.7738 × 10^−2^	3.1619 × 10^−2^	8.9809 × 10^−2^	5.3233 × 10^−2^
$f_{3}\left(\bar{p}\right)$	3.3393 × 10^−2^	5.5613 × 10^−2^	9.9995 × 10^−2^	8.6662 × 10^−2^

## Data Availability

The original contributions presented in this study are included in the article material. Further inquiries can be directed to the corresponding author(s).

## Abbreviations

The following abbreviations are used in this manuscript:

ANN	Artificial Neural Network
BVP	Boundary value problem
DoE	Design of experiment
FEM	Finite element method
GCID	Granular Computational Inverse Design
GCH	Granular Computational Homogenisation
RS	Response surface
